# Enhancing SKIN health and safety in aged CARE (SKINCARE Trial): a study protocol for an exploratory cluster-randomized pragmatic trial

**DOI:** 10.1186/s13063-019-3375-7

**Published:** 2019-05-29

**Authors:** Jan Kottner, Elisabeth Hahnel, Monira El Genedy, Konrad Neumann, Katrin Balzer

**Affiliations:** 1 Department of Dermatology and Allergy, Clinical Research Center for Hair and Skin Science, Charité-Universitätsmedizin Berlin, Charitéplatz 1, 10117 Berlin, Germany; 2 Department of Public Health and Primary Care, Skin Integrity Research Group (SKINT), University Centre for Nursing and Midwifery, Ghent University Hospital, 5K3, C. Heymanslaan, Ghent, 10 9000 Belgium; 3 Department of Biometry and Clinical Epidemiology, Charité - Universitätsmedizin Berlin, Charitéplatz 1, Berlin, 10117 Germany; 4 Sektion für Forschung und Lehre in der Pflege, Universität zu Lübeck, Institut für Sozialmedizin und Epidemiologie, Ratzeburger Allee 160, 23538 Lübeck, Germany

**Keywords:** Aged, Dermatology, Incontinence, Intertrigo, Nursing, Pressure ulcers, Skin care, Xerosis cutis

## Abstract

**Background:**

Aged long-term care receivers are affected by various adverse skin conditions like pressure ulcers, incontinence-associated dermatitis, dryness, intertrigo, and many more. Prevention of these skin problems and the provision of general hygiene and skin care activities are key areas of nursing practice. Numerous condition-specific guidelines are available and are implemented separately. On the other hand, there is huge overlap in terms of etiology, pathogenesis, and prevention of the skin conditions mentioned above. This leads to fragmented practice neglecting shared etiologies and prevention and treatment principles.

**Methods:**

The overall aims of this trial are to test the feasibility and to estimate possible effects of the implementation of a comprehensive skin care and prevention strategy targeting main nursing-relevant skin problems at the same time. A two-arm cluster-randomized controlled trial will be performed in 20 nursing homes randomly selected from the population of nursing homes of the state of Berlin, comparing skin care according to the skin care and prevention strategy with standard skin care.

**Discussion:**

It is expected that the implementation of this evidence-based skin care and prevention strategy will reduce the incidence of pressure ulcers, incontinence dermatitis, and other skin problems frequently related to care dependency. This trial will benefit individual patients and aged nursing home residents in general given the high prevalence and incidence of the addressed skin conditions. Findings of this exploratory trial may lay the foundation for a change in the development and evaluation of clinical standards and practices in general as it moves the perspective from individual conditions to a more comprehensive view on overlapping or coexisting health problems, in this case common skin conditions, in old-age long-term care receivers.

**Trial registration:**

The study is registered at the German Clinical Trials Register https://www.drks.de/drks_web/navigate.do?navigationId=trial.HTML&TRIAL_ID=DRKS00015680 (Deutsches Register Klinischer Studien, or DRKS; registration number: DRKS00015680, date of registration: January 29, 2019) and ClincialTrials.gov (registration number: NCT03824886, date of registration: January 31, 2019).

## Background

The substantial impact of age-related skin conditions, especially in old age, was recently emphasized in the latest World Health Organization (WHO) World Report on Ageing and Health [1]. The most common skin conditions in aged and care-dependent patients with a prevalence up to 99.1% in the long-term care setting are dry and vulnerable skin (including pruritus) and fungal infections with prevalences ranging from 14.3% to 64% [2, 3]. Aged and care-dependent patients are at high risk for developing pressure ulcers (PUs), skin tears (STs), intertrigo, and incontinence-associated dermatitis (IAD) [3, 4]. These are distinct clinical diagnoses, and condition-specific clinical guidelines and best practice recommendations are available [5–8]. On the other hand, these adverse skin conditions show many similarities in terms of etiology (e.g., skin fragility, immobility, and care dependency), prevention, and treatment [9].

For decades, preventive strategies implemented in clinical practice have addressed these particular skin conditions and risks separately. For example, a PU prevention policy exists in nearly every health-care institution today. Evidence-based strategies to prevent IAD, STs, or xerosis cutis are also increasingly developed. Based on local priorities and perceived needs, these recommendations are (partly) implemented or not. However, all of these distinct approaches have the same aim: to enhance and maintain skin integrity in aged and care-dependent people. Thus, proposed interventions have a lot in common: avoidance of skin-stressing insults (e.g., mechanical loading and safe handling) [7, 8], provision of skin protection and care (e.g., using protecting skin care products) [5, 7], moisturization of dry and cracked skin [6], and minimization of prolonged and repeated exposures to irritation (e.g., urine, stool, and surfactants from cleansing products) [5, 6].

Despite these shared commonalities, no overall evidence-based practice guidance exists for daily skin care in care-dependent elderly people. Facilities are challenged to implement fragmented, condition-specific guidelines, neglecting shared etiologies and prevention and treatment principles. Information overload and multiple and complex rules on the same topic have been shown to be important barriers to the implementation of evidence-based practice [10].

Skin care in general plays a fundamental role in daily nursing practice. However, the goals of providing skin care interventions do not always seem to be explicit [11], leading to a huge unexplained variability of skin care activities in daily practice [12, 13]. Research evidence suggests that current skin care is not always beneficial [14] and there seem to be misconceptions about the actual mode of action of skin care interventions [11].

Recently, an evidence-based skin care and prevention strategy was developed. The skin care strategy guides nurses through the care process starting from assessment to the allocation of targeted skin area interventions, taking into account available evidence, best practice, and guideline recommendations [15]. Implementation of the newly developed evidence-based skin health-promoting strategy will improve nurses’ knowledge, skills, and confidence in a core area of nursing practice. Additionally, it is assumed that the implementation of the skin care and prevention strategy will lead to an improvement of skin conditions, skin health, quality of life (QoL), and safety of elderly nursing home residents. However, the feasibility of implementation of this skin care and prevention strategy and its potential to influence assumed patient and intermediate outcomes have to be demonstrated before its clinical effectiveness can be examined on a larger scale.

## Objective

The overall aim of this trial is to implement a comprehensive and evidence-based skin care and prevention package that addresses common and largely avoidable adverse skin conditions in long-term care at the same time. The trial will answer the following research questions:

- Does the implementation of a skin care and prevention strategy reduce the incidence of PUs, IAD, intertrigo, and STs and the severity of dry skin in the intervention compared with the control group?
- How do different skin care regimens influence important skin barrier characteristics such as skin surface pH, stratum corneum hydration (SCH), and transepidermal water loss (TEWL)?
- Does the implementation of the skin care and prevention strategy reduce itch and pain and improve QoL?
- What are the effect sizes and intra-class correlation coefficients (ICCs) of the outcomes mentioned above?
- Is the implementation of a standardized skin care and prevention strategy feasible? What are relevant context factors?

## Methods, design, and analysis

### Design and setting

This exploratory study is a cluster-randomized pragmatic investigator-blinded parallel-group trial. The study will be conducted in a random sample of 20 out of 288 institutional long-term care facilities of the federal state of Berlin, Germany. Inclusion started April 2019 and the last nursing home resident will complete the study  March 2021.

### Nursing homes and participants

#### Eligibility criteria

The inclusion criteria at the institutional level are that (1) participating nursing homes must express a clear commitment to implement the skin care and prevention strategy if assigned to the intervention group, (2) participating nursing homes must have a valid PU prevention standard/algorithm in place, (3) only nursing homes providing written commitment to adherence to the trial procedures regardless of the outcome of randomization will be included, and (4) nursing homes have to have a minimum bed size of *n* = 70.

The inclusion criteria at the resident level are (1) living in the nursing home at the time of data collection, (2) age of at least 65 years, (3) “care degree 2 or higher” according to the German code book (Sozialgesetzbuch, or SGB) XI, and (4) written informed consent (from the resident or a legal representative). Residents at the end of life or with any dermatological condition or skin infestation requiring dermatological treatment or with known intolerance to any possible irritating product ingredients (e.g., urea or lactate buffer) or known hypersensitivity to any product ingredients (e.g., sorbid acid or cetylstearyl alcohol) will not be considered eligible.

### Interventions at the nursing home level

PU and IAD prevention and basic hygiene and skin care interventions are routinely delivered in German nursing homes. Therefore, nursing homes conducting PU prevention or other protective skin care activities (or both) according to “usual practice” will serve as the control group.

In the interventional nursing homes, a newly developed skin care and prevention strategy will be implemented on top on the existing care standards [15]. The skin care and prevention strategy will be delivered by nurses and consists of following components. Based on a comprehensive assessment, skin care will be provided. Condition-specific strategies for cleansing and the use of leave-on products will be given, including guidance on frequency of conduct/application and required product characteristics. For example, xerotic skin areas (e.g., extremities and feet) will be cleansed once daily or less often in the case of severe dryness, and lipophilic leave-on products, including humectants, will be applied at least twice daily or more often if needed. Personal care products will be used as long as they meet required product characteristics. If products are not available, a basic lipophilic emulsion (Lipophile Harnstoff Creme 5, Neues Rezeptur Formularium (NRF) 11.129) [16] will be delivered. Skin areas exposed to urine or feces (or both) will be cleansed after each incontinence episode by using mucosa-friendly mild cleansers, and a skin protection product that may include various product types (e.g., polymers) will be applied. If no products are available, a basic skin protectant (Weiche Zinkpaste DAB, NRF 11.21) [16] will be delivered and applied. Personal preferences of the residents will be taken into account during clinical decision making. The skin care and prevention strategy contains clear guidance when to involve medical experts. The components of this strategy will be adapted to local requirements as necessary.

This skin care and prevention strategy will be implemented by a combination of multiple strategies based on the normalization process theory [17], a socio-psychological middle-range theory which has been proven to be an effective framework for guideline implementation in nursing practice [18]. In addition, existing research evidence on the most relevant implementation barriers and facilitators [19–21] will be taken into account. Also, attention will be given to the four-phase model proposed by the German Network for Quality Improvement for implementing the National Expert Standards [22] because it is particularly relevant to the German nursing context.

### Strategies to improve adherence to intervention protocol

All implementation steps are designed to maximize adoption. Throughout the trial, continual communication with nursing home managers in either study group will be held to prevent early loss of clusters. Study visits to the nursing home wards conducted for data collection will maintain awareness and communication between caregivers and the study team. Because nursing home residents live in the institutions, there is a low risk that they will not be available at study visits. Before any inclusions, kick - off meetings will be held in the participating nursing homes. Study background and procedures are explained and the importance of following the study protocol is emphasized. A key project leader and additionally a “skin care team” per participating nursing home will be nominated and caregivers will be trained. The “skin care team” ideally will consist of three or four persons (e.g., quality manager, residential sector manager, and wound manager). The study team will support this team during the whole study (e.g., the study team will set goals with the project team onsite the nursing homes and will offer help at any time if problems arise) and evaluate the implementation process. Additionally, posters and flyers will be created and placed in the nursing homes for caregiver, residents, their relatives and legal representatives.

### Participant timeline

**Fig. 1:**
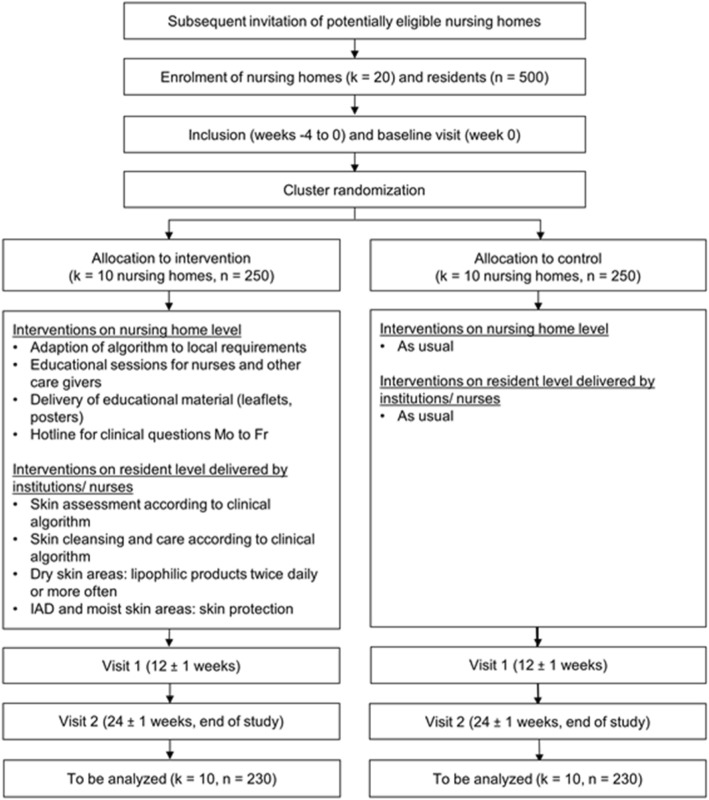
Study schedule

In total, four study visits are planned. After inclusion, residents will be followed up for 24 ± 1 weeks. Written informed consent will be obtained from the residents themselves or their legal representatives prior to study participation. All participating nursing home residents undergo a comprehensive demographic, nursing, medical, and dermatological examination. A detailed planned study schedule is shown in Fig. 1.

### Outcomes and measurements

**Fig. 2:**
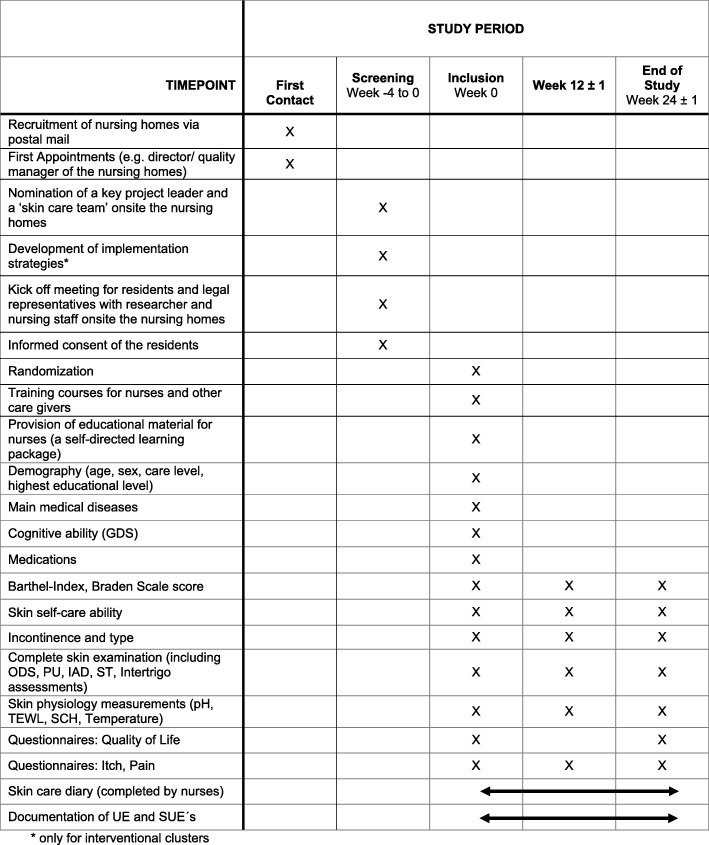
Timeline of screening and study visits

Owing to the exploratory and pragmatic nature of this trial, a broad range of outcomes will be measured. The main clinical outcomes are PU, IAD, intertrigo, and ST incidence and skin dryness per skin area. Fig. 2 shows the time points of measurement. All outcomes will be measured at baseline (week 0) and after 3 months (week 12 ± 1) and after 6 months (week 24 ± 1).

#### Dermatological examination

PU incidence and location will be classified in accordance with the National Pressure Ulcer Advisory Panel/European Pressure Ulcer Advisory Panel (2014) [23]. The IAD incidence will be classified in accordance with GLOBIAD (Ghent Global IAD Categorisation tool) [24], and coded according ICD 11 (EK02.22) and the distinction between intact and eroded skin will be classified in accordance with the *Proceedings of the Global IAD Expert Panel* [5]. Intertrigo incidence and location will be classified in accordance with ICD 11 (EK02.2), and the ST incidence and location will be classified in accordance with the International Skin Tear Advisory Panel [25].

Dry skin per skin area will be classified in accordance with the overall dry skin (ODS) score [26, 27]. The ODS score is a clinical assessment of the presence and severity of skin dryness using a five-point scale. A score of 0 indicates no skin dryness whereas a score of 4 indicates advanced skin roughness, large scales, inflammation, and cracks [26].

#### Skin physiology measurements

In addition, skin function will be assessed by measuring SCH, TEWL, and skin surface pH, which are established parameters to characterize the skin barrier function and which have been used successfully in a similar trial in this setting [28].

SCH will be measured by using the Corneometer CM 825 (Courage + Khazaka, Cologne, Germany). This measurement is based on the differences of the dielectric constant of water and other substances. With this device, only the moisture content in the stratum corneum is measured [29]. The arbitrary units range from 0 to 120, and higher readings indicate higher SCH.

TEWL will be measured with the Tewameter TM 300 (Courage + Khazaka). The probe captures the constant permeation of water through the stratum corneum in grams per hour per square meter [30]. The measuring probe contains a pair of sensors that are located at different distances from the skin surface to determine temperature and relative humidity above the skin surface. The humidity gradient between both sensors is used for calculating the TEWL. Higher values indicate a higher TEWL. Skin surface pH will be measured with the Skin-pH-Meter^®^ PH 905 (Courage + Khazaka), a planar glass electrode. pH is a measure of acidity and alkalinity of a solution and it indicates the concentration of the hydrogen ions in an aqueous solution [31]. Reference values of human skin have been reported to range from 4 to 6 [32, 33].

The skin surface temperature of the skin areas will be recorded as well. It will be measured with the Skin-Thermometer ST 500 (Courage + Khazaka) in degrees Celsius.

#### Resident-reported outcome measures

QoL in residents without cognitive impairment will be assessed with the WHO - Five Well-being Index [34] and QUALIDEM [35] for residents with dementia. The German version of the WHO - Five Well-Being Index questionnaire published by the WHO in 1998 will be used. Scores range from 5 (all the time) to 0 (never) for a total of five items. Simple questions will be asked regarding well-being in the previous two weeks (e.g., “In the last two weeks … I was happy” or “… I was relaxed”). The sum scores range from 0 (indicating the lowest well-being) to 25 (indicating the highest well-being). A cutoff score of not more or equal than 13 is recommended.

The QUALIDEM assessment is a dementia-specific QoL instrument that is carried out by the nursing staff and that allows a proxy-based QoL rating in all stages of dementia. The German version of the QUALIDEM (version 2.0) published in 2015 will be used to assess the QoL of the resident of the previous seven days [35]. If the resident is affected by a mild to severe dementia, the 37-item assessment will be used. For residents with very severe dementia the 18-item instrument will be used. To assess whether the resident is affected by mild to severe dementia or very severe dementia, the global deterioration scale (GDS) will be used [36]. GDS scores from 2 to 6 indicate a mild to severe dementia, and the 37-item assessment of the QUALIDEM tool will be used. A GDS score of 7 indicates very severe dementia, and the 18-item QUALIDEM tool will be used.

Itch will be assessed with the 5-D Itch scale [37]. The score of the 5-D Itch scale ranges from 5 (no pruritus) to 25 (most severe pruritus) and contains five items measuring pruritus over the previous two weeks. Pain will be assessed with a numeric rating scale by residents without cognitive impairments and with a verbal rating scale by residents with cognitive impairments [38].

#### Baseline assessment and covariates

Demographic variables (age, sex, care level, and highest educational level), main medical diseases, and medications will be obtained from medical records by trained study assistants. Data regarding skin self-care ability, incontinence and type will be obtained from the medical records or, if possible, via interview of the nursing home residents or the caregivers.

Functional assessments using the Braden scale and Barthel - Index will be conducted. The Braden scale is a six-item pressure ulcer risk assessment score with scores ranging from 6 (high-pressure ulcer risk) to 23 (no-pressure ulcer risk). The Barthel - Index measures physical function related to the daily activities using 10 items (e.g., washing and toilet use, eating and mobility or incontinence). Scores range from 0 (very dependent) to 100 (not dependent) [39].

All residents will be interviewed regarding their skin care habits. Data will be documented in structured source documents by trained study assistants. Variables for assessing skin care routine will be (1) washing habits, (2) showering habits, (3) bathing habits, (4) moisturizing habits of the face and body, (5) hair care, and (6) shaving habits. For all variables, the frequency, skin areas, used skin care products, and the residents’ ability to apply skin care by themselves will be documented. In case of cognitive impairment of the resident, the respective nurse will be asked or the information will be obtained from the medical records.

The residents or their caregivers will be asked regarding the date of the last visit by physician/specialty in week 0. If possible, the following information will obtained from the medical records:

1. Is there a documentation of pressure ulcer risk in place?
2. Are skin assessment results documented?
3. If applicable, the type of pressure ulcer preventive mattress or seating cushion;
4. The repositioning interval if bedfast;
5. Seating hours if not able to stand up independently;
6. Off-loading of heels if exposed to high risk of pressure ulcer;
7. Skin care interventions provided (cleansing and leave-on products);
8. Use of incontinence products and types;
9. Incontinence skin care products.

To assess the degree of implementation of the skin care package and possible moderating or mediating effects of context factors, further quantitative and qualitative data will be gathered at resident, staff, and institutional levels for process evaluation [40]. The protocol of this mixed methods study embedded in the trial will be reported separately elsewhere.

### Randomization and recruitment

A 1:1 simple random generation of the allocation sequence of nursing homes to intervention (*n* = 10) or control group (*n* = 10) will be undertaken by an independent data manager via a computer-generated random list. The allocation will be conducted by the investigator using numbered opaque envelopes (1 to 20) containing computer-generated random numbers.

Nursing homes will be randomly selected from a comprehensive list of all nursing homes in Berlin (*n* = 288). Potentially eligible nursing homes will be contacted and invited to participate by letter. If there is no response by a defined deadline, the next randomly selected nursing home will be invited. If applicable, baseline characteristics (e.g., number of beds) of non-responders and reasons for non-participation will be listed in order to estimate a possible selection bias. In the case of small institutions with fewer than 70 beds/residents, the next randomly selected institution will be invited.

Recruitment of the nursing home residents and baseline measurements will be completed prior to random allocation of the nursing homes. Posters and flyers will be placed in public areas in the nursing homes to inform residents and relatives as a first step. The recruitment onsite the nursing homes will be carried out in close cooperation with a nominated key project leader of the participating nursing home. The key project leader will inform possible residents and hand out information leaflets or letters. Interested residents or their legal representatives will be contacted by the study team personally and will be invited to participate in the study.

### Blinding

One group of investigators and study nurses administers the interventions and communicates with participating nursing homes. A second independent group of outcome assessors (including dermatologists/residents of dermatology) will complete the dermatological assessments while blinded to the allocation. The data manager and trial statistician will be blinded during data management and statistical analysis.

### Sample size calculation

Because the trial is exploratory in nature, a primary outcome is not defined. The sample size is determined such that the power of statistical tests is sufficiently high for each of several binary and continuous outcomes under realistic assumptions. According to the federal state government, there are 288 nursing homes in Berlin. According to the latest statistics (2015), the total number of nursing home residents in Berlin is 28,299. Given a mean of 100 residents per institution and a participation rate of 25%, the expected total number of residents is 25 per participating institution (500 in total). Taking into account a drop - out rate of 8%, each participating nursing home contributes about 23 residents to the trial. If m = 10 nursing homes are included in each study arm and given an ICC of ρ = 0.02 [41] and an incidence for the skin conditions of around p_C_ = 50% per annum (p.a.) (29.3% in 6 months) in the control group, the two-sided Z-test for difference in proportions at a level of α = 0.05 has a power of more than 80% if in the experimental group skin condition worsens in less than p_E_ = 29.8% p.a. (16.2% in 6 months). The power calculation for the clustered binary outcome was carried out using the method described in Ahn et al. [42]. For metrical outcomes such as the ODS score, the two-sided t-test can detect a minimal effect size (Cohen’s d) of d = 0.33 with a power of 80% at a significance level of α = 0.05. Again, an ICC of ρ = 0.02 is assumed. Therefore, the detectable effect sizes are relatively small for all outcomes. Since the effects of the experimental skin care regimen are expected to be larger, the study is not underpowered for each variable.

### Statistical analysis

All statistical evaluations will be conducted by using the statistical programming language R and IBM SPSS statistics (IBM Corporation, Armonk, NY, USA). Variables will be described at the cluster (nursing homes) and individual level on an intention-to-treat basis. A two-level analysis will be conducted for all outcomes. For comparisons of event rates in the control and experimental groups, we will use appropriate generalized mixed-effects models. Furthermore, for all outcomes, ICCs will be calculated providing a basis for future sample size estimation in a subsequent confirmatory trial. The two-sided level of significance is α = 0.05. All *p* values will be considered exploratory, no adjustment of the level of significance will be performed, and no interim analysis is planned. The data collected for process evaluation will be analyzed descriptively. The main aim of the trial is to provide a solid basis for a subsequent confirmatory trial.

### Analysis population

The statistical analysis will be run on the basis of the intention-to-treat principle. At the cluster level, the study population includes all nursing homes which have been randomly assigned. At the individual level, it contains all residents of the randomly assigned nursing homes who meet the inclusion criteria and who have passed the baseline visit. Nursing home residents who stay for longer than 2 months outside the nursing homes (e.g., due to hospital admission), die, or leave the nursing home during the study period will be analyzed until that time point (early termination).

### Trial advisory board

A trial advisory board has been implemented. On January 14, 2019 a face-to-face meeting was conducted with all advisory board members listed in Table 1. The design, conduct, feasibility, and aims were discussed with all advisory board members, and the study protocol was revised and improved on the basis of the expert recommendations. During the trial, the advisory board will oversee the study progress (including recruitment), review results of the monitoring reports, and provide recommendations on possible safety issues.

**Table 1:** Trial advisory board members and affiliations

Name	Roles and affiliations
Dimitri Beeckman	Professor of skin integrity and clinical nursing and visiting professor at Örebro University (Sweden), Royal College of Surgeons in Ireland (Ireland), and University Centre for Nursing and Midwifery, Ghent University, Belgium
Lisette Schoonhoven	Professor of nursing science, University Medical Center, Utrecht, the Netherlands
Ulrike Blume-Peytavi	Vice director of the Department of Dermatology and Allergy, Charité-Universitätsmedizin Berlin, Germany, and director of the Clinical Research Center for Hair and Skin Science at the Department of Dermatology and Allergy, Charité-Universitätsmedizin Berlin, Germany
Ursula Müller-Werdan	Director of the Evangelic Geriatric Center, Research Group Geriatrics, Charité-Universitätsmedizin Berlin, Germany
Andreas Büscher	Professor of nursing science and scientific director of the German Network for Quality Development in Nursing, Osnabrück, Germany
Gabriele Meyer	Professor of health and nursing science, Department of Health and Nursing Science, Martin-Luther University Halle, Wittenberg, Germany
Ingeborg Simon	Patient representative and member of the senior advisory council, Berlin, Germany

### Data management

A paper case report form (pCRF) file will be created for the study. Data (informed consent process and consent retrieval; demographics; medical history; clinical examination; measurements of skin functional parameters; clinical scores; occurrence of PU, IAD, xerosis cutis, ST, and intertrigo; compliance; delivery and return of the skin care diary; and delivery of products if applicable) will be recorded in written form during the study visits. An electronic case report form (eCRF) will be developed by the Charité Coordinating Center for Clinical Trials at the Charité-Universitätsmedizin Berlin (KKSC). Data from the pCRF will be extracted and entered into an eCRF. Data management and handling of data will be conducted in accordance with the trial-specific data management plan and the International Conference on Harmonisation (ICH) guidelines.

Data entry will be performed by the trial site personnel. According to a pre-defined query process, changes to data entries in the eCRF (if any) will be made at the site by the qualified trial site personnel. The eCRF will have an audit trail with appropriate functionality for data capture, tracking, and documentation of any queries or changes. Electronic signatures will be used to verify the data and identify the person entering or changing the data.

#### Data monitoring

Monitoring of the study is the responsibility of the investigator and will be undertaken by an independent monitor from the KKSC. The monitor is responsible for reviewing the progress of the study and for verifying adherence to the protocol as well as compliance to ICH Good Clinical Practice and also for handling any problems that arise. The monitor will visit the center before, during, and after completion of the study to ensure that the study is conducted, recorded, and reported in accordance with the protocol.

#### Harms

This is neither a drug nor a medical device study. The used or provided skin care products (or both) may be regarded as cosmetic products according to EU Regulation Number 1223/2009 on cosmetic products. In addition, long-term care residents are affected by a variety of diseases and medical problems. Therefore, the terminology of undesirable effects and serious undesirable effects will be adopted for this trial and will be used for the documentation throughout the study.

## Discussion

To the best of our knowledge, this is the first trial implementing a comprehensive evidence-based skin care and prevention strategy in institutional long-term care. The skin care and prevention strategy was developed systematically, taking currently available evidence and expert opinion into account. Given the novelty of this approach and its complexity, first an exploratory trial is needed in order to evaluate the feasibility of this intervention package and possible trial procedures and to gain insights into potential mechanisms of change and impacts on patient-important outcomes. Trial results will have impacts on clinical research and practice: In the short term, results will (1) prove the feasibility of the intervention package and provide robust data about relevant context factors and required implementation strategies to be considered for a confirmatory evaluation of the skin care program’s clinical effectiveness and cost-effectiveness and (2) inform the protocol for subsequent confirmatory trials (e.g., in regard to the responsiveness of outcome measures and associated effect sizes, ICCs, and data collection methods).

This trial will benefit individual patients and aged nursing home residents in general. According to the latest systematic reviews and researching [2, 3], the skin conditions of interest are frequent in this population: In German long-term and geriatric care facilities, the PU prevalence is approximately 4% [43–45], the prevalence of dry skin is approximately 53% [46] and associated pruritus up to 14% [46, 47], and the prevalence of IAD ranges from 4% to 11% [48, 49]. The incidence and severity of these skin conditions will be reduced; thus, patient safety will be enhanced. The improvement of dry skin helps to prevent secondary infection and pruritus. Overall, QoL will be increased. Potential unhelpful care interventions will be avoided. Carefully developed and targeted skin care practice will reduce patients’ exposure to unnecessary and inappropriate products, thus reducing the risk of irritation, contact allergies, and other adverse effects [11]. In addition, so far, often neglected and overlooked age-related skin conditions , especially symptomatic xerosis cutis, which is an explicitly stated unmet need of aged patients [50], will be addressed.

Findings of this exploratory trial may lay the foundation for a change in the development of clinical standards and practices in general as it moves the perspective from focusing on individual conditions to a more comprehensive view on overlapping or coexisting health problems, in this case common skin conditions, in old-aged long-term care receivers. This helps to pool resources and to increase overall patient safety within health-care institutions and to increase professional competencies of nurses, who have the major responsibility in aged care.

## Trial status

This trial is registered at the German Clinical Trials Register and ClincialTrials.gov. The local ethics committee (EA1/243/18) approved the study on January 11, 2019. At the time of submission of this manuscript, the recruitment of the nursing homes had been started. The first 12 nursing homes expressed interest. Of them, six nursing homes committed to participation and were in preparation to start the study in April and June 2019. Six other nursing homes have confirmed appointments for the first information meetings.

## Abbreviations

eCRF: Electronic case report form
GDS: Global Deterioration Scale
IAD: Incontinence-associated dermatitis
ICC: Intra-class correlation coefficient
ICD: International Classification of Diseases
ICH: International Conference on Harmonisation
KKSC: Coordinating Centre for Clinical Trials at the Charité - Universitätsmedizin Berlin
NRF: Neues Rezeptur Formularium
ODS: Overall Dry Skin
pCRF: Paper case report form
PU: Pressure ulcer
QoL: Quality of life
QUALIDEM: (No original term, measurement instrument for proxy rating of quality of life in people with dementia)
SCH: Stratum corneum hydration
ST: Skin tear
TEWL: Transepidermal water loss
WHO: World Health Organization
